# Development of Pharmacist Independent Prescribing Clinics to Treat Opioid Analgesic Dependence in NHS Lanarkshire

**DOI:** 10.3390/pharmacy7030119

**Published:** 2019-08-22

**Authors:** Duncan Hill, Elizabeth Marr, Clair Smith

**Affiliations:** 1 NHS Lanarkshire, Addictions Services, Lanarkshire ML1 2TP, UK; 2 Strathclyde Institute of Pharmacy and Biomedical Science, University of Strathclyde, Glasgow G4 0RE, UK

**Keywords:** opioid analgesic dependence, pharmacist prescribing, clinic development

## Abstract

There has been an increase in opioid analgesic prescribing in general practice (GP). This is causing some concern around this contributing to dependency. NHS Lanarkshire have attempted to reduce the prescribing from GP surgeries through the development of specialised Pharmacist Independent Prescriber clinics being delivered from the practices. This article looks at the development of these services with pharmacist independent prescribers and the results from developing the services. The article aims to provide advice and recommendations for the development of other services and strategies to minimise the risks associated with Opioid Analgesic Dependence for patients.

## 1. Background

Opioid analgesics have an important role in the management of pain and treatment for patients, but they must be used cautiously and appropriately to prevent dependency.

The extent of the problem and numbers misusing opioid analgesics is lacking and to quote Laurence [1]; there is “absolutely no data” on dependence on analgesics in the UK. Traditionally, there would be specialist information published, Information Statistics Division (ISD) data and papers written by specialists—there is no data published for the UK setting.

To compound the issue and uncertainty, having reported that data is completely lacking there are anecdotal reports and evidence that the issue is widespread. “We do not know what the scale of the opioid-related harms is, but all of us see patients in this trap in almost every clinic” [2].

In 2013, the issue of Opioid Analgesic Dependence (OAD) from prescribed medications was identified as escalating in the UK [3]. Within NHS Lanarkshire, there was a sustained and continued increase in the amount of opioid analgesics prescribed, the volumes were proportionately higher than the Scottish National average [4].

The addictions service in NHS Lanarkshire was reporting a gradual but noticeable increase in patient referrals for prescribed and Over the Counter (OTC) opioid analgesics [5]. In the UK, it is possible to purchase compound analgesics which include opioids such as co-codamol (Paracetamol and codeine), Ibuprofen and codeine or paracetamol and Dihydrocodeine from community pharmacies. Several articles have been published in this topic area and have looked at either individual case studies Conroy & Hill [6], Marr & Hill [7], Hill & Stewart [8], patient characteristics as they enter treatment services [5] and misuse of OTC medications [9,10,11,12]. There is high level social deprivation in Lanarkshire and there are higher dependence patterns in the areas of higher deprivation [13]. There was a randomized controlled trial done across two sites (Grampian and East Anglia) where there was some evidence that pharmacists had a positive effect on prescribing opioid painkillers. [14].

The issue of increased opiate prescribing in NHS Lanarkshire had been raised at an NHS board level and the need for action had been identified by doctors and pharmacists but there was a lack of services able to deliver a clinical input for this, and not many patients were being treated to address the issue (either by General Practice (GP), pain services or addiction). Nationally, there has been an increase in opioids prescribing and prescriptions and an acknowledgement that clinicians perceive an issue that needs to be identified, quantified and then addressed. Part of the perception is GPs are prescribing these opioids but they could have been commenced in secondary care but have not been reviewed (possibly due to non-attendance) and then continued to be prescribed with no review.

NHS Lanarkshire agreed to develop a potential intervention to prevent the issue of OAD and attempt to reduce the increased prescribing and prevalence of OAD and to provide methods of breaking the cycle in GP surgeries. Addiction services are ideally placed to develop this service and guide any ongoing developments once set up due to their knowledge and continued experience of treating patients with a history of opioid misuse. It was agreed that the pharmacists would only look at the painkillers and any associated medications e.g. Non-Steroidal Anti Inflammatory Drugs (NSAID) and not any other health conditions whilst in this trial phase. This meant there was limited safety concerns around prescribing but the pharmacists have access to all lab result and clinical information to base prescribing decisions on—e.g., kidney function for NSAID. The effectiveness of medication introduced was assessed at each appointment and medication was discontinued if it was not producing the desired clinical effect.

## 2. Aim

To introduce addiction-based pharmacist independent prescribers (which are pharmacists who can legally prescribe any drug that is within their clinical competence) into a GP practice to see if a difference could be made. If successful, developing a local model in NHS Lanarkshire to address the rising issue of OAD. The article discusses the development of a response in 2 phases, the second building on the first and developing from the experiences and learning cycle. The aim was to develop a possible service model to tackle the issue, along with development of good prescribing practices for GP surgeries and treatment options of patients already struggling with possible OAD. There was no investigation done into a similar group of patients that did not receive input so there is no comparison study data available.

## 3. Method

Initial phase of development commenced during 2015, when Addictions Services in NHS Lanarkshire decided to establish a pharmacist led clinic model for the GP surgery-based intervention targeting prescribed (OAD).

The selected surgery was at a large practice (18,500 patients) with a large number of GPs (8) located in an area of high social deprivation. The model used a medical note review together with a consultation with the pharmacist providing advice and recommendations including further referrals and changes to prescriptions, but did not involve prescribing. The method utilized the Opioid Risk Assessment Tool (ORAT). This was added to the surgery patient record system and helped to identify patients falling into selected criteria that may be indicative of risk of OAD, including from the number of prescriptions generated in a set time period.

In the second phase, at the end of 2016, it was agreed that addictions services could further develop the earlier work to extend the use Pharmacist Independent Prescribers (PIP) clinics into GP practices.

The development saw the service use 2 PIPs; each PIP was associated with an individual surgery and delivered one clinical session weekly. The ability of the PIPs to prescribe was to be used, with GP surgeries agreement.

Two experienced substance misuse PIPs offered to undertake the sessions and 2 different GP surgeries in NHS Lanarkshire with above average prescribing of opioid analgesics were approached one with high levels of co-codamol 30/500 mg tablets (paracetamol 500 mg codeine 30 mg) prescribed, the other high levels of dihydrocodeine. It was decided to offer the PIP clinics from the GP surgeries to avoid potential barriers of stigma and travelling to addictions service premises. An additional advantage of this model is the ability for the PIP to be viewed as part of the team within the surgery and interact with the surgery staff including the GPs.

Part of the agreement with the board and for funding was that the experience was to be used as learning and developing experience with plans of how the service was best operated and can be delivered/rolled out wider using other PIP.

For the initial phase, the surgery patient list was screened. The ORAT tool identified a large number of patients with potential OAD from the number of prescriptions (and quantities) prescribed in a 3-month period.

This data was analysed with access to Vision (the record system utilised in the practice) to identify patients with a greater risk of OAD due to co-morbidities.

For appropriate patients, an invitation letter to attend an opioid use review was sent (administration support was facilitated by the surgery). The clinics were 4 h long and allowed 12 patient appointments (a mixture of 15 min return appointments and 30 min new patients appointments), this allowed time for further patient identification and administration.

Attendance at clinic was generally poor but issues rose with administration organising and issuing clinic invitations (Table 1).

Recommendations were made for all patients in respect to their medication irrespective of attendance. A second invitation was sent if the patient failed to attend first appointment.

Only one referral to addiction services was made from the patient group, this was for alcohol use and not analgesics, which where only being used at a low level. The patient acknowledged their main issue was alcohol and the consultation moved focus from opioids to alcohol.

Several patients were already attending specialist clinics for either pain clinic or orthopedics at the hospital (e.g., one patient was waiting for back surgery following an incident 4 years previous, but needed to reduce BMI to a level before surgery would be considered but they were struggling to achieve targeted BMI as pain prevented exercising and weight loss).

3 patients were referred to the clinic for review by the surgery (both administration and GP). From these 2 attended and were reviewed, one referred to addictions for alcohol use, the other stepped down to co-codamol 8/500 from 30/500 but with a small supply of the stronger tablets for acute exacerbations and asked to speak to employers health and safety regarding work practices and recommended to attend.

In the second stage of development a number of results were achieved in the first 12 months including:

### 3.1. Patient Selection

Patients were selected in these surgeries by the use of surgery staff referral (including GP) and the ORAT. A review of the patient list by a GP was useful for an initial cohort selection, and ORAT was not always as effective as a GP had amended the dose for patients who appeared to over order. 

Learning points in relation to this are the coding of the diagnosis in the patient notes needs to be carefully and correctly inputted. After the initial patient list were exhausted it is important to check all patients on the identified medicine, some patients can be rapidly discounted but this practice allowed some further patients to be identified that the surgery are/were unaware of.

### 3.2. Initial Paperwork

#### 3.2.1. Invitation Letters

Invitation letters are valuable to call patients for the appointments and set some expectations of attendance. It has been beneficial for the template letters to be added to the surgery correspondence list so patient details can be added directly providing an accurate letter and a full audit trail. It was felt that the surgery reception staff assisting with letters and posting were a great help with this aspect.

The learning point is that many others within the practice can help and make the process run smoothly and the complete process does not rely on the PIP.

#### 3.2.2. OAD Assessment Tool

The OAD assessment tool is a “starting” point for discussion and acts as an Aide Memoir, but the use of open questions and encouraging the patient to describe their “journey” is more beneficial in information gathering. The experience of the 2 PIPs involved in the pilot phase felt that the individual discussions were more beneficial although for the initial clinics, it was helpful to have the Aide Memoire to focus the consultations and ensure everything was captured. The PIPs felt the tool should be reworded removing the word “dependence” as a gentler approach and terminology may be beneficial as many patients were unaware of their issue.

### 3.3. Pain Diary/Resources

The use of a Pain Diary for the patients to complete is beneficial, for some patients, as this allows the patient to record information such as what medication they have taken and note feelings etc. An example of one used was the NHS Greater Glasgow and Clyde pain diary. The use of some helpful leaflets was also beneficial and provided patient information on matters such as “Understanding Pain” and “Self-Management of Pain”.

Other leaflets such as the referral leaflet for physiotherapy and the chronic pain support group were also highlighted as being of assistance.

### 3.4. Surgery Prescribing

It was important for the repeat prescribing function to be deactivated when the invitation letter was sent. This encourages the patients to attend the clinic and prevents them obtaining repeats without have a discussion with the PIP.

When the repeat function was not switched off, the patients did not attend for review. When asked why they said they felt they were not given a reason to attend.

Some patients say they felt they were being penalised when this happened and it needs to be handled appropriately to prevent offence which needs to be balanced with the need to attend for a review.

For the prescribing to be controlled correctly it is important that all the prescribers within the practice are aware and need not to prescribe unless this has been approved by the PIP. If this is not controlled well, patients can play the system and also prescribers against each other. The addition of a prompt (“flash note”) to the computer record system was a helpful reminder for other prescribers that they should not be prescribing for this patient without confirming suitability of doing so with the PIP first.

Having the clinics within the practice also allowed the staff to see the PIP regularly and actively seek advice and support for other patients.

### 3.5. Patient Categories

There appears to be four categories of patients attending OAD clinics.

1. Happy on medicines, resistant to change and unwilling to try suggestions.
2. Happy on medicines, see no problem with use and want to continue but willing to try different suggestions.
3. Happy on medicines but see the problem, willing to try different suggestions.
4. Unhappy on medicines, unsure how to change but willing to try suggestions.

What was encouraging was that this was a fluid state and some patients previously reluctant to change did start to make progress and address their issues, demonstrating that OAD follows the transtheoretical cycle of change [15], akin to other forms of dependency treatment.

### 3.6. Results

Various changes and results have been obtained; these are included in Table 2.

Prescriber 1 reviewed 240 patients and had appointments with 140. Prescriber 2 reviewed 225 patients and had appointments with 125.

Both prescribers discharged a number of patients after review as the patient did not appear to be misusing the prescribed medications. Some patients had minor changes implemented via a telephone consultation, or through a letter advising them of the change (with an invitation to discuss face to face if required).

It can take several appointments and control of prescribing to get some patients into a position where they are accepting of the issue they have and bringing back under control.

**Figure 1:**
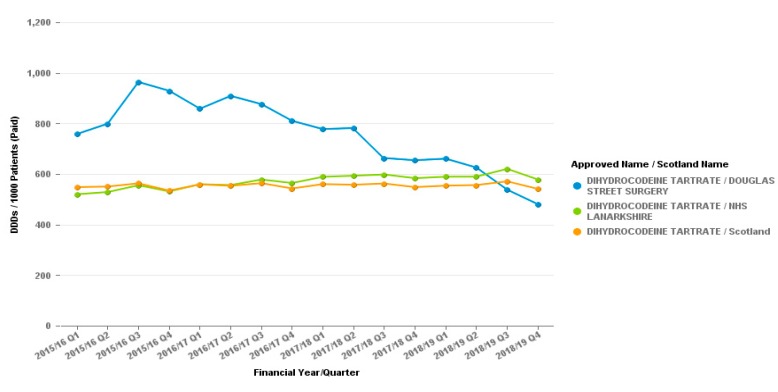
Dihydrocodeine (All preparations) Prescribing from Douglas Street, Hamilton practice. Items per 1000 patients.

Prescribing data from the period from the individual practices can demonstrate further the successes of the work. In the Hamilton practice where dihydrocodeine was targeted, it was observed that volume prescribed decreases over the time period. The volume prescribed per patient was higher than both Hamilton and NHSL at the start of the period but is now below the NHS Lanarkshire average and is much closer to the Hamilton locality average, opposite to the gradual increases shown for these areas. This is represented in Figure 1.

**Figure 2:**
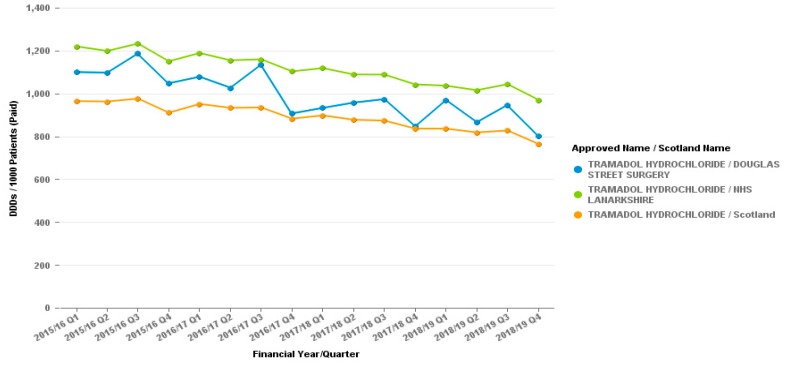
Tramadol (All preparations) Prescribing—Douglas Street Surgery, Hamilton. Items per 1000 patients.

**Figure 3:**
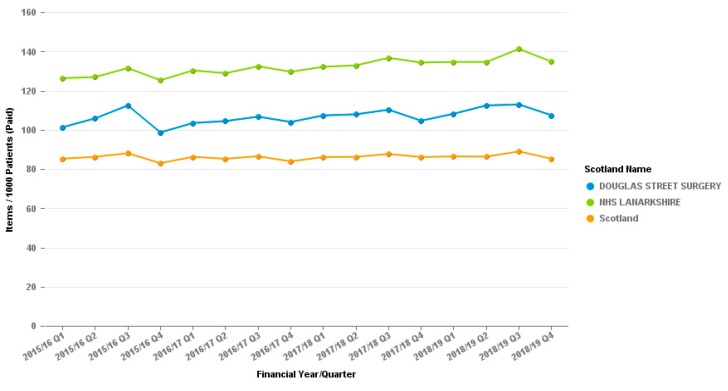
Co-codamol (All preparations) Prescribing, Douglas Street Hamilton. Items per 1000 patients.

The reduction in dihydrocodeine prescribing appears to have no effect on other opiate prescribing as seen by graphs for the tramadol or co-codamol prescribing from the surgery, as demonstrated in Figure 2 and Figure 3.

The graph for co-codamol cannot be put into the Daily Dose Units measure which the tramadol and dihydrocodeine are displayed in as it is a combination product and cannot be quantified in the same way.

**Figure 4:**
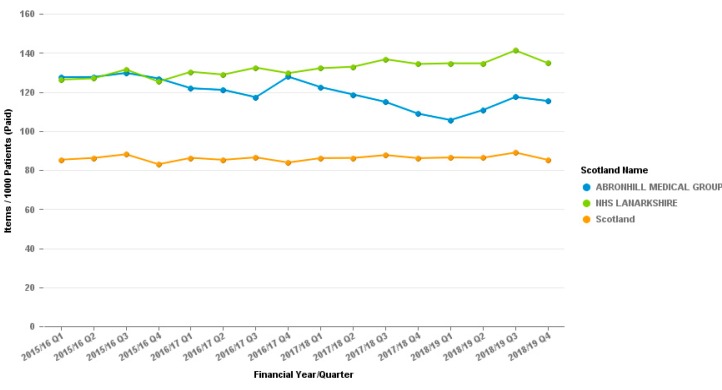
Co-codamol 30/500 (all preparations) Prescribing. Abronhill, Cumbernauld. Items per 1000 patients. The graph for co-codamol cannot be put into the Daily Dose Units measure which the tramadol and dihydrocodeine are displayed in as it is a combination product and cannot be quantified in the same way.

In the Cumbernauld GP practice the reduction of co-codamol was clear, and was much greater that the local and national prescribing data (Figure 4).

**Figure 5:**
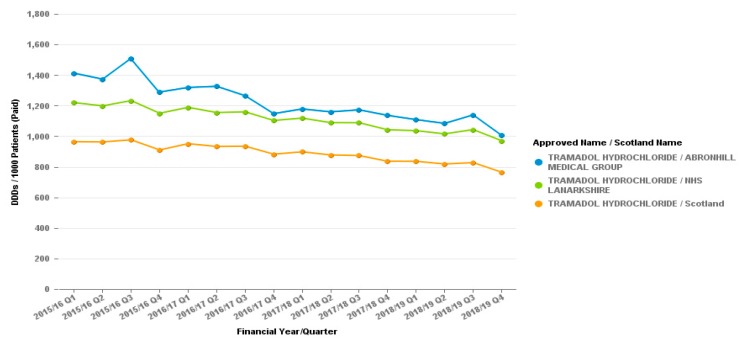
Quantity of tramadol prescribed from the GP practice in Cumbernauld. Tramadol daily dose dispensed per 1000 patients.

The Cumbernauld GP practice also looked at the prescribing of Tramadol over the same period, checking the prescribing of the alternative to Co-codamol had not increased, Figure 5 clearly demonstrates the reduction on tramadol prescribing too.

### 3.7. Changes to Medication

Changes to patient medications have been generally successful but prescribers need to be realistic about what can be achieved.

This included rationalised prescribing of opiates to a single formulation, then being able to introduce reducing regimens and other changes e.g., to weekly dispensing.

This is an area where discussions with the other prescribers within the practice is essential. The plan needs to be continued and progressing even if the PIP is not there. There needs to be clear instructions and a plan for treatment and reduction her than reverting to the previous prescribing and re-introducing the issue of poor control.

### 3.8. Other Medications Prescribed

No other medications were prescribed at the clinics other than the opioid analgesics.

When reviewing other medicines prescribed to patients by the practice, the prescribers reported a large number of the patients at the clinics were prescribed large amounts of benzodiazepines and many are on antidepressants. Both PIP also reported to have seen some patients prescribed gabapentinoids.

### 3.9. Referrals

All patients were encouraged to self-refer and attend physiotherapy. One prescriber reported all patients they had seen had been/were currently referred to physiotherapy services, however none reported to experiencing a benefit and as a result it is difficult to get these patients to re-engage with physiotherapy.

Other alternatives referred to include pain support groups, chiropractor and Pain Scotland (a peer support group).

Only one patient was encouraged and self-report to addiction services but the prescriber liaised with this service to co-ordinate the care and treatment provided.

### 3.10. Other Comments

The pain clinic was keen to engage with one of the PIPs, and actively referred a patient to the PIP to optimise the patient’s treatment with opiates before adding any other medications to the treatment prescribed.

## 4. Discussion

In the initial stage, the practice is large and has a high prevalence of potentially dependent patients receiving opioid analgesics, therefore the potential numbers to be reviewed, referred and consulted with was high and the selection of the most at risk was challenging.

There were a high number of failures to attend the reviews; all patients failing to attend the first invited clinic were offered a second appointment for review. This is despite sending 2 invitations to attend the clinic, many patients still failed to attend. There were a variety of reasons identified for patients not attending—these included anxiety re seeing someone new, misunderstanding the level of education of a pharmacist, being unwilling to see there was an issue, unwilling to change current situation either through denial of potential issues of belief status quo will remain if do not engage.

Several of the recommendations suggested were not implemented or addressed and others were reversed within 2–3 months of the recommendation being implemented, this may be to the number of patients in the surgery, regular GPs not implementing the changes, locums not sure of process, ease of changing patient back (repeating previous prescriptions) and other reasons.

The benefits seen included testing the format of the clinic which can be developed further but it allowed the initial tools for use in the clinic to be developed.

This phase tested the tool used for selection to the clinic and provided experience in the use of this combined with the patients records on the GP system (Vision) which including repeat prescribing options.

In the second phase, the reviews were offered from 2 GP surgery sites using 2 PIP who work for addiction services. As the services are established, the prescribing and control is introduced and patient numbers are increasing. Patient care remains the priority to the range of therapeutic interventions and referrals as demonstrated.

The paperwork and processes continue to be refined which over time will provide a useful example for other prescribers to get involved and deliver the same service elsewhere in NHS Lanarkshire as an attempt to tackle the issue of dependence to opioid analgesics.

Any changes to other medications were made as per local formulary and were reviewed at the same appointments reviewing the opioid analgesic to assess benefits, side effects and dose adjustment. There were no medication changes to those concerned with pain and dependency to opioids e.g., the groups of medication altered would include anti-inflammatories, opioids and antidepressants. There were no reports of adverse reactions to the medication and in general the introduction of new medications e.g., NSAIDs had a positive effect on reducing patients opioid analgesic need.

## 5. Conclusions

There is existing guidance on the treatment of pain with opiates and treatment of OAD. Current trends do not show a decrease in prescribing. This pilot study demonstrates a reduction in prescribing is possible with patient satisfaction not being compromised. Engagement with the patient is paramount. Even when resistance is present there is still room to improve outcomes by reducing opiate load and increasing functionality. The learning points from the initial phase were that the surgery used was too large to get a maximum effect. The non-prescribing approach, which relied on recommendations and changes, was not as efficient at providing the required changes and it requires a high level of support, feedback, and ongoing adherence to plans and changes.

ORAT on its own did not provide sufficient information for the determination of OAD, but in combination with the GP recording system (Vision) utilised a more robust selection process was developed to screen patients with for their OAD risk. The ORAT tool works as a pre screener, Vision adds more details but face to face with a patient and the completion of a screening form assists with patient selection.

Patients attending the reviews on the whole were happy with the process and consultation, some reported to being surprised this has not been done previously. Some patients were concerned about their medication being stopped abruptly, but when the reason for the invitation was explained and they had been reassured the purpose was not to stop the medication but make it more effective and appropriate by review they were agreeable.

From the evidence presented it can be seen that PIP involvement reduces the number of prescriptions for opiates in GP practice. The recommendation is that it is possible to introduce PIP led clinics for OAD within GP surgeries which have less stigma than attending an addictions service.

Development of guidance on good prescribing practice (Table 3) and treatment options (Table 4) after identification of an individual with opioid dependence was another positive outcome that is able to be utilised by all services.

The guidance developed encourages a consistency of approach and prescribing which should strengthen the message and reduce any exploitation of differences from different prescribers/practices. The CDC guidance gives comparisons of doses and recommendations of principles but it was felt that a stepwise approach was helpful to all practitioners in their care of individual patients.

A part of the success of the pilots was facilitated by the pharmacists prescribing and controlling the opioid analgesic prescribing for the patients identified. The process additionally built therapeutic relationships with the PIP and patient and encouraged active participation in reviewing and reducing opioid use.

The development of different treatment options which can be offered to patients not only helps standardise the offering but encourages the patient to select the treatment strategy best suited. The treatment options are displayed below in Table 4.

Prior to the establishment of the new clinic model, services had concerns that introducing a service addressing OAD would create an unknown, but potentially large number of referrals to specialised addictions services with no additional staff, in reality from our experience NHSL there were very few referrals from the two clinics (which were predominantly alcohol misuse) to addiction services, indicating that this is potentially an unwarranted concern.

From our experience, these are some of our thoughts and recommendations on what we would do if starting again from no service available:

1. No other models were functioning at the time of commencing this work, but if starting again there are several services and health boards that have now started to offer services to address opioid prescribing and OAD. We would seek their advice and support to develop a service that would be beneficial. Ask if they would be willing to share resources and documentation, but adapt as necessary for local use (but remember to acknowledge others work and development).
2. We would use a PIP who has an interest in treating opioid analgesic dependence but has an understanding of working with patients with addiction/substance misuse. The PIP will need to be resilient and may need to work with patients over a long period of time and achieve gradual but slow progress depending on patient’s response and attitude to treatment changes.
3. We would start in a manageably sized GP practice with committed GPs and administration staff.
4. We recommend that the OAD service prescribes for the patients; do not rely on recommendations of changes for GPs or administration to be made.
5. We would recommend working together with the surgery and request that the individual undertaking the clinic is responsible for all the opioid analgesic prescribing for the patients attending the clinic.
6. Screening tools do not offer the full patient identification means, there is a need to possibly use an OAD risk screening tool, but it is more important to have access to clinical records and a face to face consultation to determine fully the current situation with regards to the patient and address issues arising from the complete picture.

## 6. Future Plans and Development

Whilst this study was undertaken within the current service and as a result had no cost attached if it was to be rolled out to include a wider cohort of practices there would need to be additional funding. The agreement for funding phase 2 was dependent on developing a service and sharing a learning experience so a service could be offered elsewhere in NHSL from different GP practices.

The development and learning have been utilised in developing the “teach and treat” type model being used in NHSL.

The treatment and clinic model work and are planned to be rolled out wider to increase the changes in opiate prescribing whilst maximising patient care. Both PIPs are willing to support others in the delivery of the service and have participated at a Prescribing Support Pharmacists development session as well as allowing others to shadow clinics.

## 7. Limitations

These are only small clinics and examples of work being done and as a result with further expansion and increased numbers services will further develop and there will be further improvements and recommendations on treatment and care.

This is the experience in only one health board area and experiences in other areas may be different and only by expansion and comparison of results would the models be tested.

In the future, it may also be beneficial to have self-referral for patients who identify themselves of potentially having OAD and a means of patient referral from community pharmacy for patients they may identify as having a potential issue with dependence. Some GPs and reception staff were ideally placed for this referral to the service.

## 8. Ethics

As this was a service development and review no ethics were required. Patients were all consented to the appointments and consultations with the PIPs. All data and references included in the article are anonymous to ensure confidentiality.

## Figures and Tables

**Table 1:** Appointments and attendance at GP surgery Opioid Analgesic Dependence (OAD) clinic.

	Number of Clinics	Appointments Offered	Appointments Attended	New Patients Invited	Comments
September 2015	3	25	8	25	
October 2015	2	17	2	4	
November 2015	1	7	1	2	Invitations not sent (GP referred patient attended)
December 2015	2	17	1	7	
January 2016	1	8	0	6	Invitation not sent
February 2016	1	8	3	0	

**Table 2:** Pharmacist Prescriber results.

	Prescriber 1	Prescriber 2
Attendance	2 out of 3 people attended appts—some take repeated attempts Only 2 patients who have refused to engage	
Prescription changes (quantity)	When people come to appts usually made a difference in some way—at the very least change the amount dispensed to correspond with the actual amount taken per day E.g., instead of getting 100 tabs and taking 2 tablets 3 × daily, they get 168	Pharmacist amended repeat prescription quantity to fit dosage in 15 patients
Rationalisation of prescribing	Ten patients that have had genuine conditions that have meant rationalising scripts only. Pharmacist has 3 that have rationalised all their medication review process.	Pharmacist has rationalised prescribing of opiates to one form for 4 patients. Pharmacist has removed opiate from repeat of 6 patients as only being ordered occasionally
Reductions	Pharmacist had 20 patients that have reduced their intake of opiates by managing their dose taking better and conservative lifestyle changes	Pharmacist has reduced dosage of opiate for 11 patients
Physiotherapy referral	Fourteen have been referred to physiotherapy	Seven patients referred to physiotherapy
Other referrals	Eight patients that have liaised with GPs with other issues raised and referrals needing done	Three patients referred to pain clinic
		Ten patients referred to pain Scotland support group
		Three patients referred to Addiction Services (2 for alcohol, 1 for heroin). Pharmacist continue to work with them in the practice to reduce their opiates)
Other therapy initiation SSRI	Two patients have required initiation of an SSRI when tramadol dose reduced significantly	Pharmacist commenced 3 patients on SSRIs
Other therapy initiation Analgesics	Six patients that have added in NSAID which has resulted in reduction in opiates	Pharmacist commenced 4 patients on simple analgesia to reduce opiate dose

**Table 3:** 6 Key Principles and Actions for good prescribing practice for opioid analgesics.

1. Remove “REPEAT” ordering for all analgesics containing opioids. Prescriptions should only be produced on “ACUTE” ordering. If “REPEAT” ordering is to be used it is recommended to keep this to a maximum of 3 issues before review.
2. Minimum ordering intervals to be added to all opioid prescriptions taking into account the maximum dose and intended duration of prescription, e.g., for a 28 day prescription, the minimum re ordering interval should be 25 days.
3. Maximum prescription length of 28 days or quantity of 112 tablets per instalment, whichever is the lesser amount.
4. Stop prescribing of modified release/slow release formulations, use normal instant release preparations (with the exceptions of morphine and oxycodone).
5. Treatment review dates to be added to patient’s files and used. If preferred “Stop” dates can be used. Reviews determine ongoing need for opioid analgesia and ensure correct level being prescribed. Key recommendations 1 month post hospital discharge and 3 monthly regular reviews.
6. Specify dosage frequency on prescription. Use the exact dosing instructions to the medication, rather that the use of “as required” or “when necessary” as regular dosing can be more beneficial than ad hoc.

**Table 4:** Treatment strategy options.

Theme	Option	Comments
Tapered Reduction pathways	Change to lower strength combined analgesics e.g., Codydramol 30/500 reduced to Codydramol 20/500 (paracetamol and dihydrocodeine) Sequential dose reductions—reduction by one tablet every 14 to 28 days. Most cases starting at 8 tablets daily should be reduced to cessation where possible within 9 months If require introduced simple analgesics to control any residual pain e.g., paracetamol with regular dosing.	More than one type of reduction may be used in each individual’s case.
Change to prescribed opioids	If presenting with an OTC dependency issue (commonly codeine based either with paracetamol or ibuprofen).	
Review pain control and requirements in relation to medications prescribed or purchased OTC	If the pain is not controlled consider changes to analgesics required (preparation, dose or frequency).	Patient’s medications may not be exclusively prescribed and consider the use of OTC or substances obtained elsewhere (e.g., diverted prescriptions from friends/family) concomitantly).
Change to Opioid Substitution Therapy		Considered when patients are showing a number of aberrant behaviours and there is a definite diagnosis of dependence to OAD.
Treatment should be reviewed after a maximum period of 4 weeks after the change to monitor if this has been effective in alleviating the pain and beneficial to the patient.		Remember the dose needs to be appropriate for the patient, and dose titration may be required to address the analgesic requirements.
